# Erosion Potential of Commercial Fluoride‐Free Mouthwashes on Dental Hard Tissues

**DOI:** 10.1111/idh.70065

**Published:** 2026-04-08

**Authors:** Astrid C. Valdivia‐Tapia, Adam B. Kelly, Anderson T. Hara, Jaime Aparecido Cury, Antonio Pedro Ricomini‐Filho, Frank Lippert

**Affiliations:** ^1^ Department of Biomedical and Applied Sciences Indiana University School of Dentistry Indianapolis Indiana USA; ^2^ Piracicaba Dental School University of Campinas São Paulo Brazil

**Keywords:** erosive tooth wear, fluoride‐free, mouthwash, pH

## Abstract

**Purpose:**

Mouthwashes often present acidic pH, which raises concerns about their potential contribution to erosive tooth wear. This study evaluated the erosive potential of commercial fluoride‐free mouthwashes using a screening method.

**Methods:**

A convenience sample of 81 different mouthwashes was evaluated for their erosion potential (International Organization for Standardization, ISO/FDIS 28888:2013). This method measured the pH changes (ΔpH) of 25 mL of calcium phosphate solution (baseline pH 5.05 ± 0.05; mean ± standard deviation) once the test mouthwash was added (0.25 mL). Three citric acid/citrate buffers were used, with the maximum allowable ΔpH based on the strongest buffer. Each mouthwash was tested four times, and mean values, standard deviations, and the correlation/regression between the initial and final pH were calculated.

**Results:**

Mouthwashes presented a baseline pH range of 3.00–9.47. The reference solutions resulted in ΔpH of 0.58 ± 0.00, 0.89 ± 0.00, and 1.20 ± 0.01, respectively. None of the mouthwashes showed ΔpH higher than the strongest standard. Several unexpected results were observed: (i) nine mouthwashes with initial pH above 5.00 caused a pH drop when added to the screening solution; (ii) one mouthwash with initial pH 4.27 caused a pH drop to 4.17; and (iii) one with initial pH 4.93 caused a pH increase to 6.18. The correlation and regression values were: *r* = −0.81/*r* ^2^ = 0.65 (*p*‐value < 0.001).

**Conclusion:**

Most mouthwashes showed no erosive potential, but unexpected pH shifts in over 10% highlight the need to refine testing methods for fluoride‐free products.

**Clinical Significance:**

Some fluoride‐free mouthwashes may contribute to dental erosion due to low pH. Identifying these products helps clinicians make safer recommendations for daily use.

## 1 Introduction

Erosive tooth wear is a condition that has dental erosion as the primary etiological factor [1]. Dental erosion is a process that can lead to dental structure loss due to exposure to acids of extrinsic or intrinsic nature. For instance, mouthwashes are extrinsic agents that may present erosive potential depending on the chemical factors, such as pH, buffering capacity, and the acid type [2, 3, 4]. Several types of mouthwash are available worldwide [5, 6, 7]. These are individual/home use oral products that can be sold with or without a prescription. Depending on their formulation and purpose, they may have exhibit therapeutic or cosmetic functions. Ideally, mouthwashes should present benefits for oral health while avoiding adverse effects on the oral soft and hard tissues when used according to the manufacturer's recommendations.

The American Dental Association (ADA) states that mouthwashes can be formulated with acidic, neutral, or alkaline pH, with permissible pH values ranging from 3.0 to 10.5. For mouthwashes with a pH below 5.5, the American National Standards Institute (ANSI)/ADA Standard 116 calls for further demonstration of product safety [8]. This can be achieved through demineralization testing, erosion assays, or other scientifically appropriate methods to ensure that such formulations do not risk dental hard tissues [8]. Given the broad variability in formulations and the increasing number of mouthwash products on the market, there is a pressing need to systematically assess their potential to contribute to dental erosion.

Previous studies have examined only a limited number of these products, leaving a significant gap in the literature regarding the overall safety of commercially available mouthwashes [9]. Therefore, this study aimed to evaluate the erosive potential of a wide range of fluoride‐free commercial mouthwashes on dental hard tissues using the ISO 2813:2014 standard and to explore its potential application as a practical screening tool. It was hypothesized that fluoride‐free commercial mouthwashes with acidic pH values would exhibit a higher erosive potential on dental hard tissues than those with neutral or alkaline pH. The experimental approach followed standardized protocols based on the International Organization for Standardization (ISO) guidelines, ensuring the reliability and comparability of the results obtained [10].

## 2 Methods

### 2.1 Experimental Design

A convenience sample of 81 different fluoride‐free mouthwashes (FFM) was purchased in grocery, pharmacy, and variety stores in Indianapolis, IN, USA. The International Standard ISO/FDIS 28888:2013; Dentistry—Screening method for erosion potential of mouthwashes on dental hard tissues was used as a methodology [10]. This method is based on measuring the [*‐*]; pH changes of a calcium phosphate solution (pH = 5.05 ± 0.05; mean ± standard deviation) once the test mouthwash was added. Three reference solutions, citric acid/citrate buffers, varying in expected pH decreases (0.5, 0.7, and 1.0) once added to the calcium phosphate solution, were included. The maximum allowable decrease in pH is 1.0, corresponding to the expected pH change of the strongest buffer. Initial pH measurements of the undiluted mouthwashes were conducted, followed by measuring said pH changes. Each mouthwash was tested in quadruplicate.

### 2.2 Samples

Eighty‐one different FFM were purchased in August 2022 in Indianapolis, IN, USA. Only FFM available in ‘brick‐and‐mortar’ stores, but not those available online, were purchased. One of each grocery and variety store chain, as well as one of each pharmacy chain, was visited. No repeat purchases were made; for example, the same product available in a grocery store was not purchased again in a pharmacy. The rinses were coded with capital letters (A–CC; Table 1).

**TABLE 1:** ISO method results. pH Values found after adding the test solution to the screening solution.

Name	ISO method					
	Code	Initial pH control/mouthwash	A pH screening solution	B pH Screening solution + test solution	Difference A‐B	SD
Citric Acid 1%	1	3.60	5.00	3.80	1.20	0.01
Citric Acid 0.25%	2	3.68	5.00	4.11	0.89	0.00
Citric Acid 0.07%	3	3.77	5.00	4.43	0.58	0.00
Close UP Mouthwash with Calcium cinnamon	A	5.73	5.00	5.14	−0.14	0.00
Sea salt oral rinse	B	4.48	5.05	4.73	0.32	0.01
Colgate Optic White High Impact White Advanced	C	5.14	5.00	4.66	0.34	0.01
Colgate Peroxyl	D	3.98	5.06	5.01	0.05	0.00
Oral B Gum Detoxify	E	4.96	5.06	5.04	0.02	0.00
Crest Pro‐Health Bacteria Guard	F	4.13	5.06	4.71	0.36	0.00
Crest Pro‐Health Clinical	G	3.34	5.06	4.92	0.15	0.00
Ultimate Essential MouthCare. Eco dent	H	8.89	5.06	7.21	−2.15	0.00
Listerine Freshburst	I	4.14	5.06	4.34	0.73	0.01
Pre Brush Dental Rinse	J	6.77	5.04	5.28	−0.24	0.01
Listerine edition coconut & lime blend	K	4.20	5.03	4.42	0.61	0.00
Oral B Breath Purify	L	7.96	5.01	5.27	−0.26	0.00
Thera Breath Dentist formulated whitening fresh breath	M	4.96	5.06	4.82	0.24	0.01
Colgate Total Whole Mouth Health	N	4.51	5.04	4.76	0.29	0.00
Listerine Ultra Clean Zero alcohol	O	4.03	5.05	4.43	0.63	0.00
Cepacol Antibacterial multi‐protection mouthwash	P	7.00	5.05	5.38 4.97	−0.33	0.00
Perio Brite Complete Oral Care Natural Mouthwash	Q	4.55	5.05		0.09	0.00
Crest Pro‐Health Intense	R	4.20	5.05	4.74	0.31	0.01
Hello Activated Charcoal	S	7.18	5.04	5.33	−0.29	0.00
Jason Halthy mouth Tartar Control cinnamon clove	T	3.00	5.04	4.38	0.66	0.01
Thera Breath Dentist formulated fresh breath	U	7.22	5.04	5.55	−0.51	0.00
Parodontax active gum health Mint	V	3.89	5.02	4.51	0.51	0.01
Biotène dry mouth oral rinse	W	6.60	5.02	5.62	−0.60	0.01
ARC turns up the bright	X	5.28	5.03	4.69	0.34	0.00
Crest Pro‐Health Clean Mint multiprotection	Y	5.01	5.01	5.00	0.01	0.00
Mouthwash up & up	Z	5.27	5.02	5.00	0.02	0.00
Dr. Tichenor's All‐Natural Peppermint Mouthwash Concentrate	AA	5.21	5.00	5.01	−0.01	0.00
Antiseptic Mouthwash antigingivits antiplaque	AB	4.27	5.00	4.17	0.83	0.00
Hello Peace, out, plaque	AC	4.28	5.00	4.35	0.65	0.01
Listerine Original	AD	4.18	5.00	4.27	0.73	0.00
Lavoris Fresh Breath Mouthwash	AE	4.57	5.00	5.00	0.00	0.00
Oral B Dry mouth	AF	6.26	5.00	5.19	−0.19	0.00
Plax soft mint flavour mouthwash	AG	4.93	5.04	6.18	−1.14	0.00
ARC fresh breath mouth rinse	AH	4.96	5.03	5.02	0.02	0.00
Antiseptic Mouthwash up &up	AI	4.28	5.00	4.31	0.69	0.01
Oral B Mouth Sore	AJ	5.39	5.01	4.72	0.30	0.00
Tom's Natural Fluoride‐free Wicked Fresh	AK	4.16	5.01	4.55	0.46	0.03
Listerine Cool Mint	AL	4.25	5.07	4.32	0.75	0.00
The Natural Dentist Healthy Gums	AM	3.40	5.05	3.98	1.07	0.00
Thera breath periodontist formulated Healthy gums oral rinse	AN	5.77	5.03	5.04	−0.01	0.00
Oral B Dry mouth	AO	6.14	5.01	5.10	−0.09	0.00
Swan Mouthwash fresh mint	AP	5.30	5.01	4.99	0.02	0.00
Crest Scope mouthwash rince‐bouche	AQ	5.34	5.04	5.02	0.02	0.00
Family Wellness Blue Mint Mouthwash	AR	5.08	5.01	4.82	0.19	0.00
Crest all fresh, no stress Scope All day	AS	4.95	5.01	5.00	0.01	0.00
Desert Essence Tea Tree Oil Spearmint	AT	3.03	5.04	4.22	0.82	0.01
Jason Healthy power smile brightening	AU	3.31	5.05	4.82	0.23	0.01
Lumineux Oral essentials	AV	4.41	5.00	4.96	0.04	0.00
Jason Total Protection Sea salt mouthrinse	AW	5.40	5.05	5.41	−0.36	0.01
Tea Tree Therapy Mouth Wash	AXE	5.75	5.01	5.18	−0.17	0.00
HPM Hydrogen Peroxide Mouthwash	AY	4.79	5.00	4.98	0.02	0.01
Bubble Gum Kid's Spry Mouth wash	AZ	6.74	5.06	5.59	−0.53	0.00
Jason Healthy power smile brightening	BA	5.39	5.04	5.06	−0.02	0.00
Swan Alcohol‐free Mouthwash	BB	4.07	5.01	4.41	0.60	0.01
Orajel 2× Mouth Sores Rinse Medicated	BC	4.03	5.00	4.83	0.17	0.00
Crest Bacteria Blast	BD	5.50	5.00	4.72	0.29	0.00
Clean mint Whitening alcohol free rinse	BE	5.47	5.00	4.72	0.28	0.01
Thera Breath dentist‐formulated fresh breath oral rinse	BF	9.47	5.00	5.82	−0.82	0.01
Listerine Sensitivity Zero alcohol	BG	4.41	5.05	4.21	0.84	0.01
Splendid white whitening mouth rinse alcohol free Up and Up	BH	5.46	5.00	4.70	0.31	0.00
Crest Scope Classic	BI	5.36	5.03	5.02	0.01	0.00
BR rinse. Organic Mouthwash	BJ	4.29	5.03	4.60	0.43	0.00
Tom's Sea Salt	BK	4.31	5.00	4.79	0.21	0.00
CloSYS	BL	7.31	5.02	5.85	−0.83	0.00
Antiseptic Mouthwash antigingivitis antiplaque	BM	4.43	5.04	4.36	0.68	0.01
Crest GUM and Breath Purify	BN	4.91	5.04	5.02	0.02	0.00
Swan Antiseptic mouth rinse Original	BO	4.39	5.00	4.30	0.70	0.00
Swan Antiseptic Mouth Rinse Spring Mint	BP	4.26	5.03	4.30	0.73	0.00
Advanced Mouth Rinse	BQ	4.06	5.00	4.16	0.84	0.00
Crest 3D white brilliance	BR	5.12	5.00	4.55	0.45	0.01
Multi‐action alcohol free. Antiseptic mouth rinse	BS	3.86	5.01	4.51	0.51	0.00
BR rinse. Organic Mouthwash	BT	4.44	5.04	4.71	0.34	0.00
Guru Nanda Oxyburst Whitening Technology	BU	5.78	5.05	5.27	−0.22	0.00
PerCara Mouthwash & Gargle Refresh mint	BV	4.36	5.05	4.59	0.47	0.00
Spry Oral Rinse (Dental defence system)	BW	6.81	5.06	5.88	−0.82	0.02
Desert Essence Tea Tree Oil Whitening Plus Mouthwash	BX	3.04	5.03	4.13	0.90	0.01
Desert Essence Prebiotic Plant‐based brushing rinse Mint	BY	3.05	5.03	4.14	0.90	0.00
Smart mouth Sore zinc activated oral rinse	BZ	5.50	5.06	5.15	−0.09	0.01
Smart mouth Original	CA	5.73	5.06	5.31	−0.25	0.01
Smart mouth Clinical zinc activated oral rinse Parodontax active gum health Clear mint	CB	5.37	5.06	5.15	−0.09	0.01
	CC	3.84	5.01	4.50	0.51	0.01

*Note:* Each mouthwash was tested in quadruplicate. Values shown represent means of four independent measurements. ‘Difference A–B' indicates the pH change after addition of the test solution to the ISO screening solution. Positive values indicate a drop in pH (increased acidity), while negative values indicate a rise in pH. Shading in the ‘Code’ and ‘Initial pH’ columns represents the pH level of the solution: darker grey indicates higher pH values.(pH < 4.00; pH 4.01–5.00; pH 5.01–6.00; pH 6.01–7.00; pH > 7.01). Controls are in white colour.

Abbreviation: SD = Standard Deviation of the four measurements.

### 2.3 Preparation of Reference Buffer Solutions

Three citric acid/citrate buffers were prepared from sodium citrate dihydrate (Na_3_C_6_H_5_O_7_. 2H_2_O) and citric acid (H_3_C_6_H_5_O_7_): (1) 3.67 mM citric acid and 1.84 mM sodium citrate dihydrate (expected pH 3.60, expected pH decrease once added to screening solution 1.0); (2) 0.93 mM citric acid and 0.39 mM sodium citrate dihydrate (pH 3.68/ΔpH −0.7); and (3) 0.25 mM citric acid and 0.11 mM sodium citrate dihydrate (pH 3.77/ΔpH −0.5).

### 2.4 Screening Solution

Two stock solutions were prepared as described in the ISO/FDIS 28888:2013 manual (ISO, 2013): Stock solution A comprising 1M CaCl_2_ and 0.03 mM NaN_3_ and Stock solution B comprising 1M KH_2_PO_4_ and 0.03 mM NaN_3_. The screening solution was prepared by adding 1.266 mL of stock solution A and 0.760 mL of stock solution B to 500 mL of deionized water. The pH was adjusted to 5.05 ± 0.05 with HCl. The solution was then made up to 1 L with deionized water. This solution was prepared daily from the stock solutions.

### 2.5 Test Procedure

The test was performed at an ambient temperature (approx. 20°C). For the screening method, 25 mL of the screening solution was added to a 50 mL reaction vessel. The solution was stirred at a moderately fast rate (100 r/min) and maintained at this rate throughout the experiment. A pH‐electrode (Fisher, accumet cat. #13–620‐631), previously calibrated every four hours with standard buffer solutions of pH 4.0 and 7.0 (Fisher Chemical, Fisher Scientific), was used. The pH of the screening solution was determined. Then, 250 μL (0.25 mL) of the test material (reference buffer or mouthwash) was added to the screening solution. The reaction was terminated after a steady pH value was recorded. The test was repeated four times for each test material.

### 2.6 Data Recording and Evaluation

For each test, the pH of the test material (buffer or mouthwash), the starting pH of the screening solution, the pH of the screening solution after adding the test material, and the pH change (starting pH minus final pH) were recorded. Furthermore, the pH of each mouthwash was also determined (without dilution). A pH decrease greater than 1.0 unit upon addition of a test solution was considered critical, as defined by ISO 10993‐5 guidelines, and was used as the threshold for identifying potentially erosive mouthwashes.

Descriptive statistics were performed (means and standard deviations), and the regression and correlation between the initial pH and final pH (pH of the solution after the screening solution was added to the mouthwash) were calculated using Microsoft Excel for Mac Version 16.95.1.

## 3 Results

The present study highlighted that FFMs vary greatly in their pH (3.00–9.47), with the majority displaying a pH between pH 4.0 and 5.0. However, mouthwashes with a pH less than 5.5 are recommended to demonstrate safety using a demineralization test, erosion test, or other appropriate methods [11]. Table 1 shows the different mouthwashes with their respective codes, initial pH, pH of the used screening solution, pH of the screening solution + mouthwashes, and the mean and standard deviation of the differences. The initial pH of the mouthwashes ranged from 3.00–9.47, with 63 mouthwashes exhibiting a pH < 5.50. The results showed that eleven mouthwashes presented a pH of 3.00 to 4.00, twenty‐eight had a pH of 4.01 to 5.00, six had a pH of 4.95 to 5.05, twenty‐three had a pH of 5.01 to 6.00, seven had a pH of 6.01 to 7.00, four had a pH of 7.01–8.00, one had a pH of 8.01 to 9.00, and one had a pH > 9.01. Thirty‐two mouthwashes contained benzoic acid, 19 contained citric acid, six contained ascorbic acid, and four contained phosphoric acid.

The reference solutions resulted in pH decreases of 0.58 ± 0.00, 0.89 ± 0.00, and 1.20 ± 0.01, respectively (all mean ± standard deviation).

A moderate inverse linear correlation was observed between the mouthwashes' initial pH and the pH change after their addition to the screening solution. As shown in the regression analysis (*R* ^2^ = 0.6522), mouthwashes with lower initial pH values tended to cause a greater drop in the overall solution pH, while those with higher initial pH values resulted in smaller or even positive changes. The linear regression equation was *y* = −1.949× + 5.3221, and the correlation was *r* = −0.81 (*p* < 0.001).

Only one mouthwash (AM) resulted in a pH decrease of more than 1.00 (1.07 ± 0.00). Several unexpected pH changes were observed: (i) nine mouthwashes (C: hydrogen peroxide; M: plant extract; X, BD, BE, and BH: hydrogen peroxide and sodium hexametaphosphate; AJ: hydrogen peroxide and sodium hexametaphosphate; AR: cetylpyridinium chloride; and BR: sodium hexametaphosphate) with an initial pH > 5.00 caused a pH drop when added to the screening solution; (ii) one mouthwash (AB: essential oils) with an initial pH of 4.27 caused a pH drop to 4.17 once added to the screening solution; and (iii) one mouthwash (AG: tetrasodium pyrophosphate) with an initial pH of 4.93 caused a pH increase to 6.18 when added to the screening solution. Their ingredients can be found in Table 2.

**TABLE 2:** Information about the ingredients of FFM with specific phenomena after the test.

Code	Commercial name	Active ingredients	Other ingredients	Alcohol
C	Colgate Optic White High Impact White Advanced	Hydrogen peroxide	Water, Glycerin, Propylene Glycol, Sorbitol, Hydrogen Peroxide, Polysorbate 20, Sodium Acrylates/Methacryloylethyl Phosphate Copolymer, Phosphoric Acid, Citric Acid, Flavour, PVM/MA Copolymer, Sodium Saccharin.	—
M	Thera Breath Dentist formulated whitening fresh breath	—	Water, glycerin, polysorbate 20, sodium benzoate, PVP, Natural mint flavour, papain, D‐limonene, menthol, glucose oxidase	
X	ARC turn up the bright	Hydrogen peroxide and SH	Water, glycerin, propylene glycol, hydrogen peroxide, sodium hexametaphosphate, poloxamer 407, flavour, sodium citrate, sodium saccharin, citric acid, sucralose	—
AB	Antiseptic Mouthwash antigingivits antiplaque	Eucalyptol (0.092%), Menthol (0.042%), Methyl Salicylate (0.060%), Thymol (0.064%)	Water, Alcohol (26.9% v/v), poloxamer 407, benzoic acid sodium benzoate, Caramel	Alcohol 26.9% v/v
AG	Plax soft mint flavour mouthwash	Sodium lauryl sulfate, tetrasodium pyrophosphate	Water, sorbitol, alcohol (8.6%), tetrasodium pyrophosphate, sodium benzoate, benzoic acid, sodium lauryl sulfate, poloxamer 407, flavour, xanthan gum, sodium saccharin, blue 1, yellow 5	Alcohol 8.6% v/v
AJ	Oral B Mouth Sore	Hydrogen peroxide 1.5% /(W/v) and sodium hexametaphosphate	Citric acid, flavour, glycerin, poloxamer 407, propylene glycol, sodium citrate, sodium hexametaphosphate, sodium saccharin, sucralose, water	—
AR	Family Wellness Blue Mint Mouthwash	Cetylpyridinium Chloride	Water, alcohol (12.25 wt%), glycerin and/or sorbitol, polysorbate 80 and/or polysorbate 20, flavour, sodium saccharin, sodium benzoate, cetylpiridinium chloride, benzoic acid, blue 1	12.25%
BD	Crest Bacteria Blast	Hydrogen peroxide and sodium hexametaphosphate	Water, glycerin, alcohol (5 wt%), hydrogen peroxide, sodium hexametaphosphate, poloxamer 407, flavour, sodium citrate, sodium saccharin, citric acid, sucralose	Alcohol 5% wt%
BE	Clean mint Whitening alcohol‐free rinse	Hydrogen peroxide and sodium hexametaphosphate	Water, glycerin, hydrogen peroxide, propylene glycol, sodium hexametaphosphate, poloxamer 407, sodium citrate, flavour, PEG‐40, Hydrogenated castor oil, sodium saccharin, citric acid.	—
BH	Splendid white whitening mouth rinse alcohol‐free Up & Up	Hydrogen peroxide and sodium hexametaphosphate	Water, glycerin, hydrogen peroxide, propylene glycol, sodium hexametaphosphate, poloxamer 407, sodium citrate, flavour, PEG‐40, Hydrogenated castor oil, sodium saccharin, citric acid.	—
BR	Crest 3D white brilliance	Hydrogen peroxide and sodium hexametaphosphate	Water, glycerin, sodium hexametaphosphate, poloxamer 407, sodium benzoate, sodium lauryl sulfate, flavour, phosphoric acid, sodium saccharin, sucralose, red 33, green 3	—

*Note:* The ingredients listed are based on the product label descriptions. Exact concentrations or percentage values of individual ingredients were not disclosed by the manufacturers and are therefore not provided.

## 4 Discussion

Dental erosion refers to the chemical dissolution of tooth hard tissues due to frequent exposure to acids not produced by oral bacteria [1]. Although human saliva contains natural buffer systems that help neutralize potentially erosive acids [12], the dilution of mouthwashes in the oral cavity is minimal [13]. As a result, the initial pH of mouthwash products is largely preserved during use. Acidic oral hygiene products have previously been associated with dental erosion [14, 15]. This study aimed to evaluate the erosive potential of commercially available FFMs by assessing their initial pH values and their interactions with a standardized screening solution. The control solutions were intended to serve as comparative benchmarks for evaluating the pH‐lowering potential of test products. Specifically, they allow the classification of test solutions based on whether their ΔpH values are lower, similar, or higher than those of the reference controls. The results presented in Table 1 reflect the pH dynamics before and after adding each mouthwash to the ISO screening solution. The column labelled “Difference A–B” quantifies the change in pH, with positive values indicating a decrease in pH and negative values representing a rise in pH. This difference provides insight into each product's acidifying potential, which may indicate erosive capacity.

The findings revealed that 63 out of 81 products had a pH below the critical threshold of 5.50, suggesting a potential risk for dental erosion. Most mouthwashes showed pH values between 3.00 and 6.00, with only a few exhibiting neutral or alkaline levels. Additionally, some products caused significant pH drops upon interaction with the screening solution, with one product (AM) resulting in a decrease greater than 1.00 unit. A moderate linear correlation was observed between the initial pH of the mouthwashes and the final pH of the screening solution. However, the range and variability in pH change observed among the mouthwashes are likely influenced by their specific chemical compositions. Acids such as benzoic, citric, ascorbic, and phosphoric, identified in many formulations, serve multiple roles, including preservation, flavor enhancement, and antimicrobial function [7]. However, these acids can significantly lower the pH of the solution, increasing the erosive potential when the product is used regularly. For example, several mouthwashes with a starting pH above 5.0 still caused a notable pH drop when added to the screening solution. This suggests that ingredient interactions in solution may further impact acidity and, consequently, the risk of enamel demineralization. The presence of acidic components may explain some of the unexpected pH drops observed in products such as hydrogen peroxide or essential oil‐based mouthwashes.

Only one mouthwash (AM) exceeded the ISO‐defined limit of a 1.0‐unit pH drop (1.07), and failed to meet ISO 10993‐5 criteria. Its primary ingredients included 20% *Aloe Vera* and citric acid. However, a reference buffer of citric acid/sodium citrate caused an even greater pH drop (1.20), raising questions about test interpretation. Therefore, it could be argued that mouthwash AM did not necessarily fail the test. These findings, along with other unexpected pH changes, emphasize the complexity of commercial formulations and support the need for further refinement of the current screening methodology.

As pH alone does not fully capture erosive potential, ISO developed the standardized screening method ISO/FDIS 28888:2013 to offer a consistent, reproducible in vitro approach. The rationale for using the ISO method is to detect those that are potentially erosive. For those outside the limits (Delta pH > 1), or where questions arise, a more elaborate study should be carried out. A recent study proposed a different method to determine “the safety of low pH oral care rinse products to dental enamel” [16]. Their method is more sophisticated and clinically relevant as it involves enamel specimens that undergo a cycling procedure with changes in surface microhardness being used as the outcome measure. However, the ideal would be to carry out a previous study such as the ISO method. Another point to assess is that this method alone is not suitable as a screening method as testing the presently evaluated 81 mouthwashes would likely take several months. Another methodology used to evaluate the erosive potential of different substances is the pH‐stat method. It uses an automatic titrator that adds an acidic titrant to maintain a constant pH of a solution containing hydroxyapatite and the test agent. The necessary volume of titrator to maintain the pH is then converted to the amount of hydroxyapatite dissolved [17]. This test can also be conducted manually. The pH‐stat method may be more clinically relevant because it is a technique that studies dental erosion and incorporates hydroxyapatite crystals that provide information on its interactions with acid in erosive challenges. A balance needs to be found for a screening method that is both clinically relevant yet can be accomplished in a short time frame.

One of the limitations of this study was the variability in pH measurements, as they were conducted on different days, which may have led to slight inconsistencies despite the use of freshly prepared solutions. The complex formulations of commercial mouthwashes also resulted in unexpected pH changes that could not be fully explained. Additionally, relying solely on pH to assess erosive potential is insufficient, as it does not account for buffering capacity or ingredient interactions. While the ISO 28888:2013 method provides a standardized approach, it is time‐consuming and may lack clinical relevance compared to more advanced methods like surface microhardness testing or the pH‐stat technique. Finally, interpreting the ISO criteria can be challenging, as some test results appeared inconsistent or difficult to justify. Long‐term exposure simulations and studies incorporating saliva‐like conditions would better replicate the oral environment. The correlation between the ISO pH screening results and actual enamel erosion should also be validated to assess the utility of the ISO method as a reliable screening tool.

## 5 Conclusion

Commercial FFMs exhibit wide pH variability, with many posing a potential risk for dental erosion. Standardized ISO‐based screening is crucial to identify products that require further safety assessment under ANSI/ADA guidelines. The frequent presence of acidic ingredients and the inconsistent pH behavior upon dilution underscore the need for comprehensive evaluation beyond pH measurement alone.

## 6 Clinical Relevance

### 6.1 Scientific Rationale for Study

Given the extensive range of commercially available products, and previous research has examined only a limited subset, assessing their potential to induce dental erosion is essential. Principal findings: FFM varies greatly in pH; although, based on an ISO screening method, none of the presently tested 81 products were found to have detrimental effects on the dental hard tissues. Practical implications: Based on the ISO‐method findings of this study, none of the FFM assessed exhibited erosive potential. Nevertheless, certain anomalous behaviours were observed in some products which, despite passing the test, may suggest instability in some of their components.

## Author Contributions

A.C.V.‐T., A.T.H., and F.L. performed conceptualization; A.C.V.‐T., A.B.K., A.T.H., and F.L. performed data curation; A.C.V.‐T. and A.B.K. performed formal analysis; A.C.V.‐T., A.B.K., A.T.H., and F.L. carried out investigation and methodology drafting; A.T.H., F.L., J.A.C., and A.P.R.‐F. conducted project administration; A.T.H. and F.L. collected resources; A.T.H. and F.L. conducted supervision; A.C.V.‐T., A.T.H., and F.L. conducted validation and visualization, and writing – original draft preparation; and A.C.V.‐T., A.B.K, A.T.H., J.A.C., A.P.R.‐F., and F.L. performed writing – review and editing.

## Funding

The authors have nothing to report.

## Conflicts of Interest

The authors declare no conflicts of interest.

## Data Availability

Data sharing not applicable to this article as no datasets were generated or analysed during the current study.
